# Real-World Treatment Patterns Among Patients With Metastatic Castration-Resistant Prostate Cancer: Results From an International Study

**DOI:** 10.1093/oncolo/oyad046

**Published:** 2023-04-04

**Authors:** Pedro C Barata, Andrea Leith, Amanda Ribbands, Rachel Montgomery, Matthew Last, Bhakti Arondekar, Jasmina Ivanova, Alexander Niyazov

**Affiliations:** Department of Hematology Oncology, University Hospitals Seidman Cancer Center, Cleveland, OH, USA; Department of Internal Medicine, Adelphi Real World, Bollington, UK; Department of Internal Medicine, Adelphi Real World, Bollington, UK; Department of Internal Medicine, Adelphi Real World, Bollington, UK; Department of Internal Medicine, Formerly of Adelphi Real World, Bollington, UK; Global Value and Evidence, Oncology, Pfizer Inc., Collegeville, PA, USA; Global Value and Evidence, Oncology, Pfizer Inc., New York, NY, USA; Global Value and Evidence, Oncology, Pfizer Inc., New York, NY, USA

**Keywords:** disease management, prostatic neoplasms, castration-resistant/drug therapy, androgen antagonists

## Abstract

**Background:**

There is limited real-world evidence on how increasing use of treatment intensification in metastatic castration-sensitive prostate cancer (mCSPC) has influenced treatment decisions in metastatic castration-resistant prostate cancer (mCRPC). The study objective was to evaluate the impact of novel hormonal therapy (NHT) and docetaxel use in mCSPC on first-line treatment patterns among patients with mCRPC in 5 European countries and the United States (US).

**Methods:**

Physician-reported data on patients with mCRPC from the Adelphi Prostate Cancer Disease Specific Program were descriptively analyzed.

**Results:**

A total of 215 physicians provided data on 722 patients with mCRPC. Across 5 European countries and the US, 65% and 75% of patients, respectively, received NHT, and 28% and 9% of patients, respectively, received taxane chemotherapy as first-line mCRPC treatment. In Europe, patients who had received NHT in mCSPC (*n* = 76) mostly received taxane chemotherapy in mCRPC (55%). Patients who had received taxane chemotherapy, or who did not receive taxane chemotherapy or NHT in mCSPC (*n* = 98 and 434, respectively) mostly received NHT in mCRPC (62% and 73%, respectively). In the US, patients who had received NHT, taxane chemotherapy, or neither in mCSPC (*n* = 32, 12, and 72, respectively) mostly received NHT in mCRPC (53%, 83%, and 83%, respectively). Two patients in Europe were rechallenged with the same NHT.

**Conclusions:**

These findings suggest that physicians consider mCSPC treatment history when making first-line treatment decisions in mCRPC. Further studies are needed to better understand optimal treatment sequencing, especially as new treatments emerge.

## Teaser

This study evaluated whether treatments used in the metastatic castration-sensitive prostate cancer (mCSPC) setting influence choice of first-line treatment of metastatic castration-resistant prostate cancer (mCRPC) across a diverse, real-world patient population in five European countries and the US.

**Implications for Practice**

There is limited real-world evidence on how treatment decisions in metastatic castration-resistant prostate cancer (mCRPC) are influenced by the increasing use of treatment intensification (novel hormonal therapies and docetaxel) in metastatic castration-sensitive prostate cancer (mCSPC). The findings of this real-world study suggest that physicians in 5 European countries and the US considered previous treatments received in the mCSPC setting when making treatment decisions for their patients with mCRPC. As the use of intensified treatment in mCSPC increases and more treatment options become available in mCRPC, greater insight into optimal treatment sequencing will be required.

## Introduction

The treatment landscape for metastatic castration-­resistant prostate cancer (mCRPC) has expanded over the past 2 decades since approval of the taxane chemotherapy, docetaxel, in 2004 in the United States (US; Supplementary Fig. S1).^1-20^ Since approval in mCRPC, docetaxel and novel hormonal therapies (NHTs) are being administered earlier in the prostate cancer treatment continuum. Following the survival benefit shown in clinical trials,^21-26^ NHTs^27,28^ and docetaxel^29^ in Europe, and NHTs^30,31^ in the US, are now approved for use as intensified treatment in metastatic castration-sensitive prostate cancer (mCSPC). Although real-world US and Canadian studies show that most patients still do not receive intensified treatment in the mCSPC setting, there has been a trend toward increased treatment intensification in recent years.^32-43^

American Urological Association (AUA)/American Society for Radiation Oncology (ASTRO)/Society for Urologic Oncology (SUO), European Association of Urology (EAU), and European Society for Medical Oncology (ESMO) guidelines state that prior treatment sequencing should be considered when choosing treatment in mCRPC, and recommend a treatment with an alternative mechanism of action to previous therapies.^44-46^ These guidelines also recommend molecular sequencing when patients are refractory or non-responsive to androgen deprivation therapy (ADT) with docetaxel or NHT, to elucidate the next best course of action.^44-46^ There is evidence that molecular profiling could be used to further stratify patients with refractory mCRPC, and thus better inform treatment selection and sequencing.^47^

Real-world evidence is important to inform our understanding of current mCRPC treatment patterns. However, the few studies that have been published on treatment sequencing in mCRPC tended to consider first-line and second-line mCRPC treatment only, without consideration for how treatment intensification in the mCSPC setting could influence treatment decisions in the mCRPC setting.^48-50^ Insight into optimal treatment sequencing is becoming increasingly important with the anticipated increased use of NHTs in the mCSPC setting and as further new treatment options become available. As such, the objective of this study was to evaluate whether treatments used in the mCSPC setting influence choice of first-line treatment in mCRPC across a diverse, real-world patient population in 5 European countries (United Kingdom [UK], France, Germany, Spain, and Italy) and the US.

## Methods

### Study Design

Data from the Prostate Cancer Disease Specific Program (DSP) were utilized and subsequently analyzed for the purpose of this study. The DSP is a point-in-time, physician-­conducted extraction of medical chart data. The surveys are conducted in routine clinical practice, and the data collected describe patient demographics and clinical characteristics; and prostate cancer disease management including treatment history, the burden and impact of prostate cancer, and associated treatment effects from the perspective of the physician. The DSP methodology has been previously published and validated.^51-53^

Physicians in the UK, France, Germany, Spain, Italy, and the US reported information for their patients with metastatic prostate cancer attending a physician’s appointment between January and August 2020.

### Participants

A geographically diverse sample of physicians was identified by local fieldwork agents using physician panels and publicly available lists. All physicians self-identified as oncologists or urologists. All physicians had personal responsibility for prescribing decisions for patients with prostate cancer, and were seeing 2 or more patients with mCRPC (at time of data collection) and 2 or more patients with mCSPC per month.

### Patient Inclusion Criteria

Patients included in this study were male, ≥18 years old, currently diagnosed with mCRPC, had never taken part in a clinical trial, and were receiving systemic drug treatment for metastatic prostate cancer (any line). Patients who had orchiectomy alone were not included in this analysis. All patients who had a drug treatment for mCRPC initiated between 2016 and 2020 were included in this analysis. All patients in this analysis progressed from mCSPC to mCRPC.

### Physician-Reported Data

Participating physicians completed an attitudinal survey with questions on physician and practice characteristics. Following this, physicians completed detailed patient record forms (PRFs) for their next 4 eligible, adult patients treated for mCRPC. The number of patients per physician was limited as such to allow for a varied representation. The PRFs collected detailed information on patient and clinical characteristics, patient management, and treatment history at the time of data collection. Ethnicity was identified by physicians and was not self-identified by patients.

The Eastern Cooperative Oncology Group Performance Status Scale (ECOG) was used to assess performance status, which scores from 0 (fully active) to 4 (completely disabled).^54^

### Treatments Received

First-line mCRPC treatments were described overall and stratified by treatments received in the mCSPC setting. Patients were classed as either treated with no NHT and no taxane chemotherapy in mCSPC; treated with taxane chemotherapy in mCSPC (docetaxel, cabazitaxel, or paclitaxel); or treated with a NHT (abiraterone, apalutamide, or enzalutamide) in mCSPC. mCSPC treatment groups were allowed to overlap (ie, patients who were treated with taxane chemotherapy in mCSPC and who were treated with a NHT in mCSPC).

### Ethics

The Adelphi DSP was submitted to and obtained exemption from the Western Institutional Review Board, study protocol number AG8741.

Data collection was undertaken in line with European Pharmaceutical Marketing Research Association guidelines^55^ and as such it did not require ethics committee approval. Each survey was performed in full accordance with relevant legislation at the time of data collection, including the US Health Insurance Portability and Accountability Act 1996,^56^ and the Health Information Technology for Economic and Clinical Health Act.^57^ Data were collected in such a way that patients and physicians could not be identified directly.

### Analysis

Data were analyzed descriptively using IBM SPSS Data Collection Survey Reporter Version 6 or later (International Business Machines Corp., New York, USA). For continuous variables, we reported mean and SD, and/or median and range. For categorical variables, frequency and percentage distribution were reported.

## Results

### Patient Characteristics

Across all countries, 215 physicians (187 across 5 European countries and 28 in the US) provided data on 722 patients (606 across 5 European countries and 116 in the US) with mCRPC. Across the 5 European countries, at the time of data collection, the majority (93%) of patients were White/Caucasian, with a median (range) age of 72 (45-90) years (Table 1A). Approximately half (55%) of the patients in Europe were treated at academic medical centers and only 8% had a known family history of prostate cancer (Table 1A). Most patients (87%) in Europe were treated by oncologists and 13% were treated by urologists. Bone metastases and visceral metastases were present in 89% and 27% of patients in Europe, respectively (Table 1B). In the US, at the time of data collection, the most common ethnicities were White/Caucasian (61%) and African American (25%), with a median (range) age of 69 (50-90) years (Table 1A). Over half of patients (60%) in the US were treated at academic medical centers and 15% had a known family history of prostate cancer. Most patients (83%) in the US were treated by oncologists and 17% were treated by urologists. Bone and visceral metastases were evident in 70% and 33% of patients in the US, respectively (Table 1B). Further baseline characteristics are available in Supplementary Tables S1–S3.

### Overall Treatment Patterns Across 5 European Countries

Across the 5 European countries in this study, most patients (53%) initiated first-line mCRPC treatment in 2020. In the total analysis population (*n* = 606), NHT was the most common first-line treatment (65%; *n* = 396). Abiraterone was administered more frequently than enzalutamide as first-line treatment in mCRPC (35% [*n* = 215] and 29% [*n* = 177] of all patients in Europe, respectively). Apalutamide was administered to 1% (*n* = 4) of all patients in Europe as first-line treatment in mCRPC. Taxane chemotherapy was used as first-line treatment for 28% (*n* = 170) of patients (20% docetaxel [*n* = 124] and 8% cabazitaxel [*n* = 46]). Only 5% of patients (*n* = 30) received ADT with or without a first-generation nonsteroidal anti-androgen (NSAA) as first-line treatment in mCRPC (Table 2).

**Table 2.** First-line mCRPC treatment patterns by mCSPC treatment history across 5 European countries and the US.

	Europe^a^				US			
First-line mCRPC treatment, *n* (%)	Treated with taxane chemotherapy (*n* = 98)	Treated with NHT (*n* = 76)	No NHT and no taxane chemotherapy (*n* = 434)	Total (*n* = 606)^b^	Treated with taxane chemotherapy (*n* = 12)	Treated with NHT (*n =* 32)	No NHT and no taxane chemotherapy (*n* = 72)	Total (*n* = 116)
NHT ± ADT^c^	61 (62)	17 (22)	318 (73)	396 (65)	10 (83)	17 (53)	60 (83)	87 (75)
Enzalutamide	21 (21)	8 (11)	148 (34)	177 (29)	5 (42)	4 (13)	42 (58)	51 (44)
Abiraterone	40 (41)	7 (9)	168 (39)	215 (35)	5 (42)	11 (34)	17 (24)	33 (28)
Apalutamide	0 (0)	2 (3)	2 (0)	4 (1)	0 (0)	2 (6)	1 (1)	3 (3)
Taxane chemotherapy ± NHT ± ADT^c^	29 (30)	42 (55)	100 (23)	170 (28)	0 (0)	5 (16)	5 (7)	10 (9)
Cabazitaxel	23 (23)	6 (8)	18 (4)	46 (8)	0 (0)	0 (0)	0 (0)	0 (0)
Docetaxel	6 (6)	36 (47)	82 (19)	124 (20)	0 (0)	5 (16)	5 (7)	10 (9)
Taxane chemotherapy + NHT ± ADT^c^	7 (7)	2 (3)	1 (0)	10 (2)	0 (0)	0 (0)	0 (0)	0 (0)
Cabazitaxel	4 (4)	2 (3)	0 (0)	6 (1)	0 (0)	0 (0)	0 (0)	0 (0)
Docetaxel	3 (3)	0 (0)	1 (0)	4 (1)	0 (0)	0 (0)	0 (0)	0 (0)
Taxane ± ADT^c^	22 (22)	40 (53)	99 (23)	160 (26)	0 (0)	5 (16)	5 (7)	10 (9)
Cabazitaxel	19 (19)	4 (5)	18 (4)	40 (7)	0 (0)	0 (0)	0 (0)	0 (0)
Docetaxel	3 (3)	36 (47)	81 (19)	120 (20)	0 (0)	5 (16)	5 (7)	10 (9)
* *ADT^c^	5 (5)	14 (18)	11 (3)	30 (5)	2 (17)	7 (22)	3 (4)	12 (10)
Other	3 (3)	3 (4)	5 (1)	10 (2)	0 (0)	3 (9)	4 (6)	7 (6)
Sipuleucel-T containing regimen (no radium-223)	1 (1)	0 (0)	0 (0)	1 (0)	0 (0)	0 (0)	3 (4)	3 (3)
Radium-223 containing regimen (no sipuleucel-T)	0 (0)	2 (3)	3 (1)	5 (1)	0 (0)	0 (0)	1 (1)	1 (1)
Abiraterone + enzalutamide	1 (1)	0 (0)	0 (0)	1 (0)	0 (0)	0 (0)	0 (0)	0 (0)
Cabazitaxel + docetaxel	0 (0)	0 (0)	0 (0)	0 (0)	0 (0)	2 (67)	0 (0)	2 (29)
Diethylstilbestrol	0 (0)	0 (0)	1 (0)	1 (0)	0 (0)	0 (0)	0 (0)	0 (0)
Leuprorelin + cisplatin	0 (0)	0 (0)	0 (0)	0 (0)	0 (0)	1 (33)	0 (0)	1 (14)
Bisphosphonates	1 (1)	1 (1)	0 (0)	1 (0)	0 (0)	0 (0)	0 (0)	0 (0)
Paclitaxel + carboplatin	0 (0)	0 (0)	1 (0)	1 (0)	0 (0)	0 (0)	0 (0)	0 (0)

^a^Includes 5 European countries only: UK, France, Germany, Spain, and Italy.

^b^In Europe, 2 patients were treated with both taxane chemotherapy and NHT in mCSPC.

^c^ADT with or without a first-generation NSAA.

Abbreviations: ADT, androgen deprivation therapy; mCRPC, metastatic castration-resistant prostate cancer; mCSPC, metastatic castration-sensitive prostate cancer; NHT, novel hormonal therapy; NSAA, nonsteroidal anti-androgen; UK, United Kingdom; US, United States.

**Figure 1.**
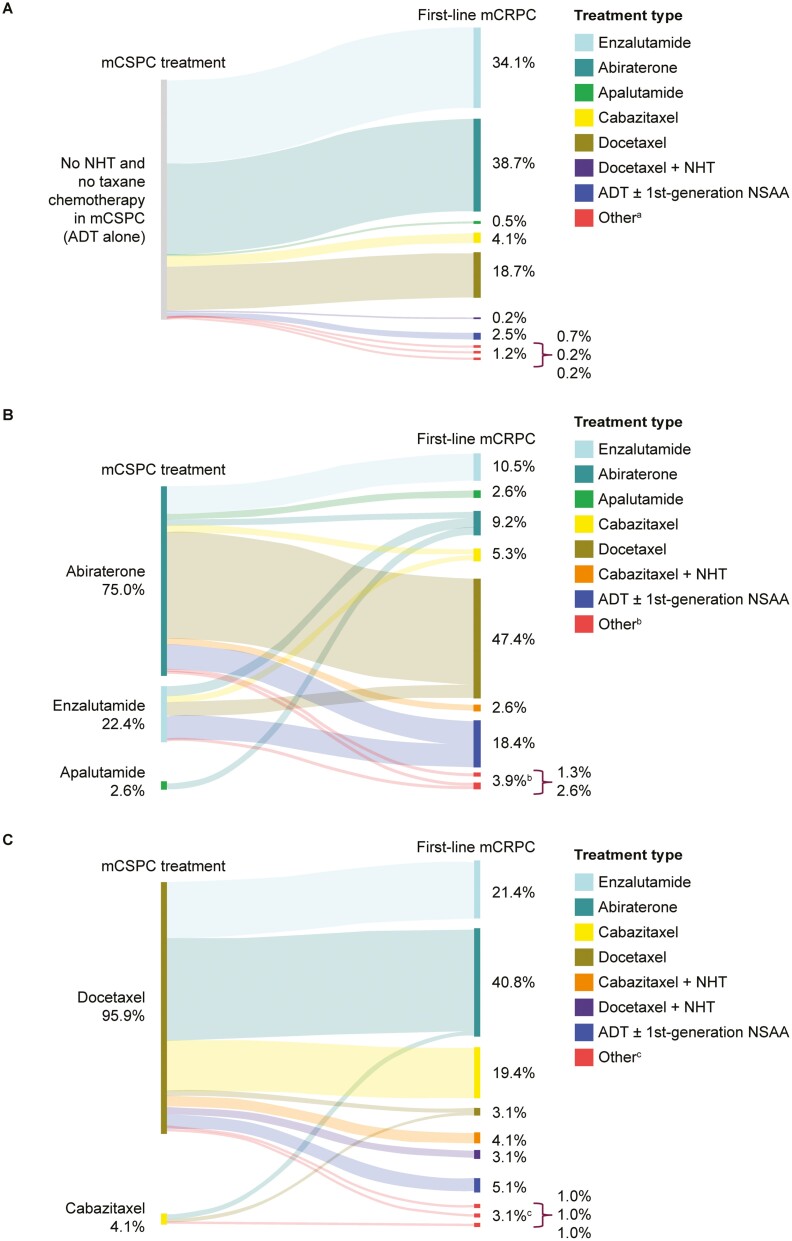
mCRPC treatment patterns among patients across 5 European countries. mCRPC treatment patterns across 5 European countries by mCSPC treatment history: (**A**) mCRPC treatment patterns among patients across 5 European countries with no NHT and no taxane chemotherapy treatment in mCSPC (*n* = 434); (**B**) mCRPC treatment patterns among patients across 5 European countries who received treatment with an NHT in mCSPC (*n* = 76); (**C**) mCRPC treatment patterns among patients across 5 European countries who received treatment with taxane chemotherapy in mCSPC (*n* = 98). Five European countries include: UK, France, Germany, Spain, and Italy. ^a^Other treatments include, from top to bottom: radium-223-containing regimen (no sipuleucel-T), diethylstilbestrol, paclitaxel + carboplatin. ^b^Other treatments include, from top to bottom: bisphosphonates, radium-223-containing regimen (no sipuleucel-T). ^c^Other treatments include, from top to bottom: bisphosphonates, sipuleucel-T-containing regimen (no radium-223), bisphosphonates, abiraterone + enzalutamide. Abbreviations: ADT, androgen deprivation therapy; CT, chemotherapy; mCRPC, metastatic castration-resistant prostate cancer; mCSPC, metastatic castration-sensitive prostate cancer; NHT, novel hormonal therapy; NSAA, nonsteroidal anti-androgen; UK, United Kingdom.

When the overall analysis population was subgrouped by mCSPC treatment history, 72% of patients (*n* = 434) received no NHT and no taxane chemotherapy. Most of these patients (73%; *n* = 318) received an NHT (mainly abiraterone or enzalutamide) as first-line treatment in mCRPC (Fig. 1A). For patients who received an NHT in the mCSPC setting (13%; *n* = 76), docetaxel was the most common first-line mCRPC treatment (47%; *n* = 36; Fig. 1B). Among patients who received an NHT in the mCSPC setting, 22% received another NHT in mCRPC (*n* = 17). Use of the same NHT drug in the mCSPC setting and as first-line treatment in mCRPC was minimal (*n* = 2). For patients who received a taxane chemotherapy (16%; *n* = 98; mainly docetaxel), the most common first-line mCRPC treatments were abiraterone (41%; *n* = 40), enzalutamide (21%; *n* = 21), and cabazitaxel (23%; *n* = 23; Fig. 1C).

Abiraterone was administered more frequently than enzalutamide as treatment in the mCSPC setting (9% [*n* = 57] and 3% [*n* = 17] of all patients in Europe, respectively). Apalutamide was administered to <1% (*n* = 2) of all patients in Europe in the mCSPC setting.

### Overall Treatment Patterns in the US

In the US, most patients (68%) initiated first-line mCRPC treatment in 2020. In the total analysis population (*n* = 116), NHT was the most common first-line treatment in mCRPC (75%; *n* = 87). Enzalutamide was received more frequently than abiraterone as first-line mCRPC treatment (44% [*n* = 51] and 28% [*n* = 33] of all US patients, respectively). Apalutamide was received by 3% (*n* = 3) of all US patients as first-line mCRPC treatment. The numbers of patients administered taxane chemotherapy (docetaxel) or ADT with or without NSAA as first-line treatment in mCRPC were similar (9%, *n* = 10; 10%, *n* = 12; respectively) (Table 2).

**Figure 2.**
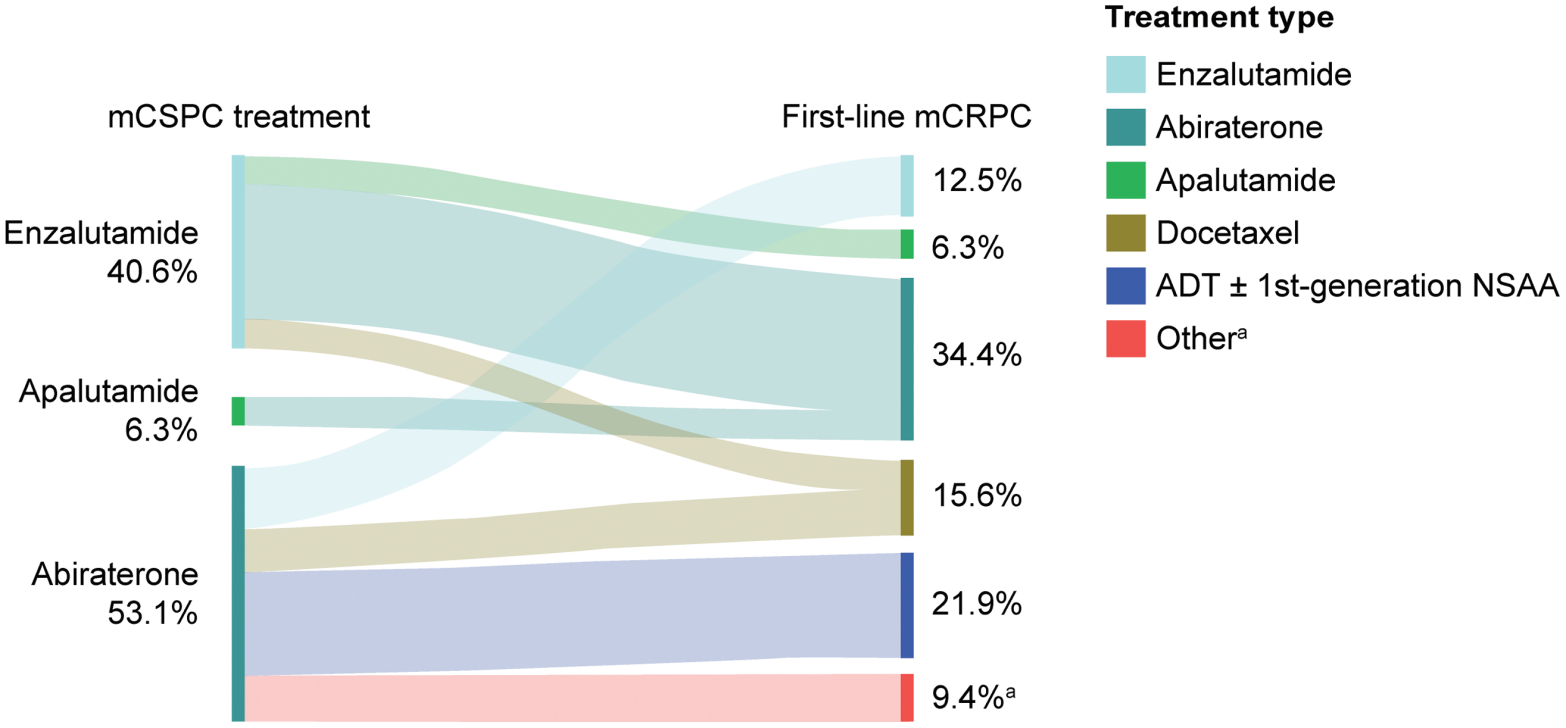
mCRPC treatment patterns among patients in the US who received treatment with an NHT in mCSPC. mCRPC treatment patterns in the US by mCSPC NHT treatment history (*n* = 32). ^a^Other treatments include: cisplatin, cabazitaxel + docetaxel. Abbreviations: ADT, androgen deprivation therapy; mCRPC, metastatic castration-resistant prostate cancer; mCSPC, metastatic castration-sensitive prostate cancer; NHT, novel hormonal therapy; NSAA, nonsteroidal anti-androgen; US, United States.

When the overall analysis population was subgrouped by mCSPC treatment history, 62% of patients (*n* = 72) received no NHT and no taxane chemotherapy. Subsequently, most of these patients (83%; *n* = 60) were administered an NHT (mainly abiraterone or enzalutamide) as first-line treatment in mCRPC. In the mCSPC setting, 10% of patients (*n* = 12) received a taxane chemotherapy (mainly docetaxel) and 28% of patients (*n* = 32) received an NHT. Among these patients, an NHT was the most common first-line treatment in mCRPC (*n* = 10, 83%; and *n* = 17, 53%; respectively). All patients treated with an NHT in the mCSPC setting went on to receive a different NHT drug as first-line treatment in mCRPC than the NHT used in mCSPC (Fig. 2).

Abiraterone was administered more frequently to treat mCSPC than enzalutamide (15% [*n* = 17] and 11% [*n* = 13] of all US patients, respectively). Apalutamide was administered to 2% (*n* = 2) of all US patients in the mCSPC setting.

### Treatment Trends by Key Patient Demographics

For first-line treatment in mCRPC, even with large differences in patient numbers, treatments were similar across ethnicities. In Europe, of White/Caucasian patients (*n* = 564), Afro-Caribbean patients (*n* = 17), and patients of other ethnicities (*n* = 25), 67% (*n* = 380), 65% (*n* = 11), and 60% (*n* = 15), respectively, were administered an NHT (abiraterone, apalutamide, or enzalutamide). In the US, of White/Caucasian patients (*n* = 71), African American patients (*n* = 29), and patients of other ethnicities (*n* = 16), 77% (*n* = 55), 72% (*n* = 21), and 69% (*n* = 11), respectively, were administered an NHT (abiraterone, apalutamide, or enzalutamide; Supplementary Table S4).

Most patients were administered an NHT as first-line treatment in mCRPC independent of whether they were treated by an oncologist or urologist. Of 529 oncologist-treated patients in Europe, 67% (*n* = 353) received an NHT (abiraterone, apalutamide, or enzalutamide). Of 77 urologist-treated patients in Europe, 69% (*n* = 53) received an NHT (abiraterone, apalutamide, or enzalutamide). Of 96 oncologist-treated patients in the US, 74% (*n* = 71) received an NHT (abiraterone, apalutamide, or enzalutamide). Of 20 urologist-treated patients in the US, 80% (*n* = 16) received an NHT (abiraterone or enzalutamide; Supplementary Table S5).

For patients in the US with Medicare or commercial insurance, most were administered an NHT (abiraterone, apalutamide, or enzalutamide) as first-line treatment in mCRPC. Of 62 patients in Medicare, 71% (*n* = 44) received an NHT. Of 41 patients with commercial insurance, 83% (*n* = 34) received an NHT (Supplementary Table S6).

In the mCSPC setting, the majority of patients in both Europe and the US were not administered an NHT or a taxane chemotherapy across ethnicities (Europe: White/Caucasian, 71% [*n* = 401]; Afro-Caribbean, 82% [*n* = 14]; other ethnicities, 76% [*n* = 19]; US: White/Caucasian, 62% [*n* = 44]; African American, 52% [*n* = 15]; other ethnicities, 81% [*n* = 13]; Supplementary Table S4).

For both oncologists and urologists, in the mCSPC setting most patients were not treated with an NHT or a taxane chemotherapy; however, the percentage difference between specialties in the US was large. In Europe, 71% (*n* = 378) of ­oncologist-treated patients and 73% (*n* = 56) of urologist-treated patients received no NHT and no taxane chemotherapy. In the US, 56% (*n* = 54) of oncologist-treated patients and 90% (*n* = 18) of urologist-treated patients received no NHT and no taxane chemotherapy (Supplementary Table S5).

For patients in the US with Medicare or commercial insurance, most were not administered an NHT or a taxane chemotherapy in the mCSPC setting (Medicare, 55% [*n* = 34]; commercial insurance, 71% [*n* = 29]; Supplementary Table S6).

## Discussion

Our study found that, across 5 European countries and the US, NHT was the most common first-line treatment in mCRPC. Other US real-world studies have shown a similar preference for NHTs over taxane chemotherapy for first- and second-line treatment.^58,59^ Consistent with guideline recommendations,^44-46^ the findings of our study suggest that physicians considered previous treatments received in the mCSPC setting when making treatment decisions in first-line mCRPC.

A US, real-world study of patients with mCRPC treated in the Veterans Health Administration (VHA) looked at 2 cohorts of mCRPC treatment, reflecting the more current and prior treatment landscapes: patients treated between 2006 and 2010, and patients treated between 2011 and 2016.^60^ The study found that first-line use of abiraterone and enzalutamide increased in the later time period, but also that docetaxel use declined from 83% to 36% of patients in favor of these therapies. Similarly, another US, real-world study using the Flatiron database demonstrated that NHTs were received by 63% of patients initiating first-line mCRPC treatment between 2013 and 2019.^59^

The PROXIMA study, an international, real-world study of treatment patterns in patients with mCRPC previously treated with docetaxel in Asia, Europe, Latin America, and other countries from November 2011 to July 2015 found that, overall, hormone therapy was more frequently used as subsequent treatment than chemotherapy.^61^ Fifty-eight percent of patients went on to receive a hormonal therapy following docetaxel. It was also found that treatment patterns were influenced by region, with hormonal therapy used more and chemotherapy used less across the 5 European countries compared with other regions. Treatment patterns were not heavily influenced by physician specialty, prior ADT duration, or Charlson Comorbidity Index score at inclusion.^61^

It is important to consider that the data collection period overlapped with the COVID-19 pandemic, with most patients in this study initiating first-line mCRPC treatment in 2020. This may have impacted preferences for oral therapies that can be administered at home, rather than treatment requiring an in-person healthcare visit such as intravenous chemotherapy. A global survey of 129 healthcare professionals across 17 different countries, including the UK, Spain, and the US, found that all institutions in the study implemented some changes in the delivery of treatment.^62^ In total, 45% of these institutions switched from systemic therapies to oral anti-cancer therapies due to the COVID-19 pandemic. This may, in part, explain the high level of NHT use seen in our study.

In our study, as in a number of claims-based analyses,^32-43^ most patients did not receive an NHT in mCSPC. Results from a physician survey linked to patient chart reviews in the US suggest that this may be due to financial considerations as well as misperceptions of guidelines, tolerability, and efficacy, some of which may be overcome with further medical education.^63^ In addition to prior treatment exposure during mCSPC, approximately half of all patients who receive first-line mCRPC treatment go on to receive subsequent lines of treatment.^58,59^ It should also be noted that more treatment options will become available in the future, such as triple therapies, PARP inhibitors, PTEN loss therapies, immunotherapies, and lutetium PSMA. Thus, there will be an increasingly unmet need for further insight into the optimal treatment sequencing for patients with mCRPC.

### Limitations

Physician selection in the DSP is a potential bias as it is influenced by willingness to take part in the study and may not be representative of the overall population of physicians treating prostate cancer. A selection bias with regard to ethnicity may also result from the participating providers, which could have been overcome by targeting providers with a greater minority population. Another limitation results from the fact that patients with more frequent visits are more likely to be included in the sample than patients with less frequent visits to their physicians. It must also be recognized that the study included only consulting patients with mCRPC; survival bias likely skews the data toward patients at an earlier disease stage and may explain the low rate of visceral metastasis. In addition, only patients who received first-line treatment in mCRPC are included in the study. Our study may therefore not fully represent the overall population of patients with mCRPC. However, the systematic approach to recruitment intends to reduce selection bias.

Although the overall sample size for this study was large, sample sizes per country and by patient demographic subgroups were small; future studies with larger sample sizes should investigate this further. Additionally, reimbursement for different medications varies widely by country and will therefore influence the treatment trends of each country studied. Reimbursement in European countries may vary by regions within countries; however, this level of analysis was not possible. Choice of mCSPC treatment may also reflect the historical lack of approved treatments. This reflects an inherent limitation of the constantly changing environment of real-world analyses. Further studies should explore time at-risk for mCSPC treatment and take historical treatment approvals into account, but our study does not have a sufficient sample size to conduct such an analysis.

Our study uses descriptive analyses only and we are therefore unable to make conclusions based on comparisons between patient groups.

### Conclusions

While additional studies with larger sample sizes are needed, the findings of our real-world study suggest that physicians consider previous mCSPC treatments when making treatment decisions in mCRPC, and that their choice of first-line mCRPC treatment is impacted by NHTs moving earlier in the prostate cancer continuum. Insight into optimal treatment sequencing will become increasingly important with the anticipated increased use of NHT in the mCSPC setting and as new treatment options become available.

## Supplementary Material

Supplementary material is available at *The Oncologist* online.

**Table 1A.** Patient baseline demographics by mCSPC treatment history across 5 European countries and the US.

	Europe^a^				US				Europe^a^ and US
	Treated with taxane chemotherapy (*n* = 98)	Treated with NHT (*n* = 76)	No NHT and no taxane chemotherapy (*n* = 434)	Total (*n* = 606)^b^	Treated with taxane chemotherapy (*n* = 12)	Treated with NHT (*n =* 32)	No NHT and no taxane chemotherapy (*n* = 72)	Total (*n* = 116)	Total (*n* = 722)
Physician specialty, *n* (%)									
* *Oncologist	84 (86)	69 (91)	378 (87)	529 (87)	12 (100)	30 (94)	54 (75)	96 (83)	625 (87)
Medical oncologist	73 (74)	66 (87)	346 (80)	483 (80)	12 (100)	30 (94)	54 (75)	96 (83)	579 (80)
Radiation oncologist	0 (0)	1 (1)	6 (1)	7 (1)	0 (0)	0 (0)	0 (0)	0 (0)	7 (1)
Clinical oncologist^c^	11 (11)	2 (3)	26 (6)	39 (6)	0 (0)	0 (0)	0 (0)	0 (0)	39 (5)
Urologist	14 (14)	7 (9)	56 (13)	77 (13)	0 (0)	2 (6)	18 (25)	20 (17)	97 (13)
Hospital type, *n* (%)									
Academic/cancer center	45 (46)	41 (54)	249 (57)	333 (55)	11 (92)	17 (53)	42 (58)	70 (60)	403 (56)
Community	53 (54)	35 (46)	185 (43)	273 (45)	1 (8)	15 (47)	30 (42)	46 (40)	319 (44)
Patient age at time of data collection, *n* (%)									
Median (range)	69.0 (49-84)	73.0 (55-90)	73.0 (45-90)	72.0 (45-90)	69.5 (60-80)	71.5 (50-83)	68.0 (56-90)	69.0 (50-90)	72.0 (45-90)
Family history of prostate cancer, *n* (%)									
Yes	11 (11)	8 (11)	30 (7)	48 (8)	2 (17)	7 (22)	8 (11)	17 (15)	65 (9)
No	79 (81)	64 (84)	377 (87)	519 (86)	10 (83)	20 (63)	60 (83)	90 (78)	609 (84)
Unknown	8 (8)	4 (5)	27 (6)	39 (6)	0 (0)	5 (16)	4 (6)	9 (8)	48 (7)
Ethnic origin, *n* (%)									
White/Caucasian	91 (93)	74 (97)	401 (92)	564 (93)	8 (67)	19 (59)	44 (61)	71 (61)	—
African American	—	—	—	—	3 (25)	11 (34)	15 (21)	29 (25)	—
Afro-Caribbean	3 (3)	0 (0)	14 (3)	17 (3)	—	—	—	—	—
Other^d^	4 (4)	2 (3)	19 (5)	25 (4)	1 (8)	2 (6)	13 (18)	16 (14)	—

^a^Includes 5 European countries only: UK, France, Germany, Spain, and Italy.

^b^In Europe, 2 patients were treated with both taxane chemotherapy and NHT in mCSPC.

^c^Clinical oncologist is a UK-specific specialty covering both medical and radiation specialties.

^d^Other ethnicities include: Asian (Indian subcontinent); Asian (other); Hispanic/Latino; Middle Eastern; and mixed race.

Abbreviations: NHT, novel hormonal therapy; UK, United Kingdom; US, United States.

**Table 1B.** Patient baseline disease characteristics by mCSPC treatment history across 5 European countries and the US.

	Europe^a^				US				Europe^a^ and US
	Treated with taxane chemotherapy (*n* = 98)	Treated with NHT (*n* = 76)	No NHT and no taxane chemotherapy (*n* = 434)	Total (*n* = 606)^b^	Treated with taxane chemotherapy (*n* = 12)	Treated with NHT (*n =* 32)	No NHT and no taxane chemotherapy (*n* = 72)	Total (*n* = 116)	Total (*n* = 722)
ECOG at time of data collection, *n* (%)									
0	15 (15)	6 (8)	74 (17)	95 (16)	2 (17)	3 (9)	9 (13)	14 (12)	109 (15)
1	60 (61)	44 (58)	264 (61)	367 (61)	8 (67)	18 (56)	44 (61)	70 (60)	437 (61)
2	19 (19)	25 (33)	78 (18)	121 (20)	1 (8)	9 (28)	18 (25)	28 (24)	149 (21)
3	1 (1)	0 (0)	15 (3)	16 (3)	0 (0)	1 (3)	0 (0)	1 (1)	17 (2)
4	1 (1)	1 (1)	1 (0)	3 (0)	1 (8)	1 (3)	1 (1)	3 (3)	6 (1)
Unknown/not assessed	2 (2)	0 (0)	2 (0)	4 (1)	0 (0)	0 (0)	0 (0)	0 (0)	4 (1)
Number of comorbidities at time of data collection									
Median (range)	2.0 (1-11)	2.0 (1-11)	2.0 (1-9)	2.0 (1-11)	3.0 (1-5)	3.0 (1-7)	3.0 (1-8)	3.0 (1-8)	2.0 (1-11)
Number of symptoms at time of data collection									
Total	97	75	429	599	12	30	72	114	713
Median (range)	2.0 (0-8)	2.0 (0-7)	2.0 (0-7)	2.0 (0-8)	1.0 (0-4)	2.0 (0-8)	1.0 (0-7)	1.0 (0-8)	2.0 (0-8)
Patients with metastases at time of data collection, *n* (%)									
Bone	83 (85)	66 (87)	390 (90)	537 (89)	9 (75)	28 (88)	44 (61)	81 (70)	618 (86)
Non-regional/distant lymph nodes	40 (41)	32 (42)	159 (37)	230 (38)	4 (33)	9 (28)	25 (35)	38 (33)	268 (37)
Visceral	33 (34)	34 (45)	99 (23)	164 (27)	4 (33)	9 (28)	25 (35)	38 (33)	202 (28)
* *Liver	15 (15)	12 (16)	33 (8)	59 (10)	1 (8)	3 (9)	1 (1)	5 (4)	64 (9)
Other	1 (1)	0 (0)	0 (0)	1 (0)	0 (0)	0 (0)	0 (0)	0 (0)	1 (0)
Disease volume at time of data collection, *n* (%)									
High	51 (52)	51 (67)	175 (40)	275 (45)	6 (50)	11 (34)	33 (46)	50 (43)	325 (45)
Low	34 (35)	17 (22)	157 (36)	208 (34)	6 (50)	12 (38)	26 (36)	44 (38)	252 (35)
Don't Know	13 (13)	8 (11)	102 (24)	123 (20)	0 (0)	9 (28)	13 (18)	22 (19)	145 (20)
Disease state at initial prostate cancer diagnosis, *n* (%)									
Metastatic disease (*de novo*)	60 (61)	48 (63)	297 (68)	404 (67)	12 (100)	21 (66)	54 (75)	87 (75)	491 (68)
Locally advanced/regional disease (recurrent)	29 (30)	15 (20)	73 (17)	117 (19)	0 (0)	6 (19)	12 (17)	18 (16)	135 (19)
Localized disease/clinically localized disease (recurrent; any risk level)	9 (9)	13 (17)	64 (15)	85 (14)	0 (0)	5 (16)	6 (8)	11 (9)	96 (13)
Gleason score at initial prostate cancer diagnosis									
Median (range)	8 (0-10)	8 (0-9)	7 (0-10)	7 (0-10)	8 (0-9)	7 (0-9)	7 (0-9)	7 (0-9)	7 (0-10)
Time from mCRPC diagnosis to first-line mCRPC treatment (days)									
Total	84	71	391	544	12	29	68	109	—
Median (range)	2.0 (0-627)	4.0 (0-1015)	4.0 (0-1556)	3.5 (0-1556)	0.0 (0-758)	8.0 (0-355)	7.0 (0-885)	7.0 (0-885)	—

^a^Includes 5 European countries only: UK, France, Germany, Spain, and Italy.

^b^In Europe, 2 patients were treated with both taxane chemotherapy and NHT in mCSPC.

Abbreviations: ECOG, Eastern Cooperative Oncology Group; mCSPC, metastatic castration-sensitive prostate cancer; mCRPC, metastatic castration-resistant prostate cancer; NHT, novel hormonal therapy; UK, United Kingdom; US, United States.

oyad046_suppl_Supplementary_Figure

oyad046_suppl_Supplementary_Tables

## Data Availability

All data (methodology, materials, data, and data analysis) that support the findings of this survey are the intellectual property of Adelphi Real World. All requests for access should be addressed directly to Amanda Ribbands at amanda.ribbands@adelphigroup.com. Amanda Ribbands is an employee of Adelphi Real World.
