# Supplementary material for: Real-World Treatment Patterns Among Patients With Metastatic Castration-Resistant Prostate Cancer: Results From an International Study
Source: Oncologist. 2023 Apr 4;28(9):e737–47. doi: 10.1093/oncolo/oyad046 (PMC10485288; doi:10.1093/oncolo/oyad046)
Supplement: oyad046_suppl_Supplementary_Figure [file oyad046_suppl_supplementary_figure.docx]

**Supplementary Figure S1.** European Medicines Agency and United States Food and Drug Administration approval dates for the treatment of mCRPC.^1-20^


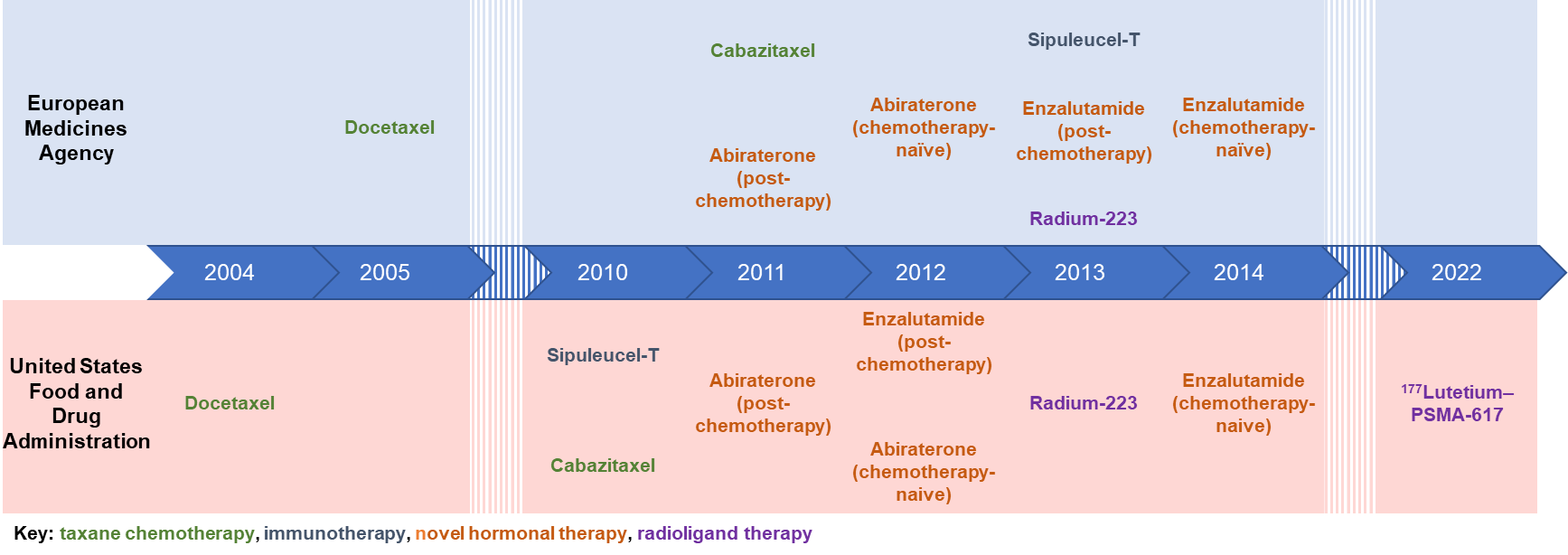


European Medicines Agency approval of ^177^lutetium–PSMA-617 is expected in the near future. PARP inhibitors olaparib and rucaparib and the immunotherapy pembrolizumab are also options for patients with mCRPC who have genomic alterations.
