# Supplementary material for: Real-World Treatment Patterns Among Patients With Metastatic Castration-Resistant Prostate Cancer: Results From an International Study
Source: Oncologist. 2023 Apr 4;28(9):e737–47. doi: 10.1093/oncolo/oyad046 (PMC10485288; doi:10.1093/oncolo/oyad046)
Supplement: oyad046_suppl_Supplementary_Tables [file oyad046_suppl_supplementary_tables.docx]

**Supplementary Table S1.** Insurance type by mCSPC treatment history across five European countries and the US.

| **Insurance type at time of data collection, *n* (%)** | **Europe^a^** | | | | **US** | | | |
| --- | --- | --- | --- | --- | --- | --- | --- | --- |
|  | **Treated with taxane chemotherapy**  **(*n* = 98)** | **Treated with NHT**  **(*n* = 76)** | **No NHT and no taxane chemotherapy**  **(*n* = 434)** | **Total**  **(*n* = 606)** | **Treated with taxane chemotherapy**  **(*n* = 12)** | **Treated with NHT**  **(*n* = 32)** | **No NHT and no taxane chemotherapy**  **(*n* = 72)** | **Total**  **(*n* = 116)** |
| Public | 88 (90) | 70 (92) | 398 (92) | 555 (92) | 9 (75) | 22 (69) | 42 (58) | 73 (63) |
| Private | 5 (5) | 4 (5) | 5 (1) | 13 (2) | 3 (25) | 9 (28) | 29 (40) | 41 (35) |
| Mixed | 1 (1) | 0 (0) | 6 (1) | 7 (1) | 0 (0) | 0 (0) | 0 (0) | 0 (0) |
| Don't know/no insurance | 4 (4) | 2 (3) | 25 (6) | 31 (5) | 0 (0) | 1 (3) | 1 (1) | 2 (2) |

^a^Includes five European countries only: UK, France, Germany, Spain, and Italy.

mCSPC, metastatic castration-sensitive prostate cancer; NHT, novel hormonal therapy; UK, United Kingdom; US, United States.

**Supplementary Table S2.** Supportive drug therapies for prostate cancer received at data collection by mCSPC treatment history across five European countries and the US.

| **Supportive drug therapies for prostate cancer received at data collection, *n* (%)** | **Europe**^a^ | | | | **US** | | | |
| --- | --- | --- | --- | --- | --- | --- | --- | --- |
|  | **Treated with taxane chemotherapy**  **(*n* = 98)** | **Treated with NHT**  **(*n* = 76)** | **No NHT and no taxane chemotherapy**  **(*n* = 434)** | **Total**  **(*n* = 606)** | **Treated with taxane chemotherapy**  **(*n* = 12)** | **Treated with NHT**  **(*n* = 32)** | **No NHT and no taxane chemotherapy**  **(*n* = 72)** | **Total**  **(*n* = 116)** |
| Not receiving any supportive drug therapies | 24 (24) | 19 (25) | 142 (33) | 184 (30) | 4 (33) | 3 (9) | 25 (35) | 32 (28) |
| Non-opioid analgesics | 36 (37) | 22 (29) | 151 (35) | 209 (34) | 0 (0) | 12 (38) | 26 (36) | 38 (33) |
| Opioid analgesics | 44 (45) | 33 (43) | 123 (28) | 199 (33) | 1 (8) | 11 (34) | 5 (7) | 17 (15) |
| Steroids | 23 (23) | 27 (36) | 78 (18) | 127 (21) | 1 (8) | 2 (6) | 1 (1) | 4 (3) |
| Anti-emetics | 24 (24) | 25 (33) | 57 (13) | 106 (17) | 1 (8) | 6 (19) | 5 (7) | 12 (10) |
| Radioisotopes for bone metastases (eg samarium, strontium, rhenium) | 2 (2) | 2 (3) | 6 (1) | 10 (2) | 0 (0) | 1 (3) | 1 (1) | 2 (2) |
| Bone-targeting agents (eg denosumab, zoledronate) | 31 (32) | 20 (26) | 112 (26) | 163 (27) | 7 (58) | 18 (56) | 24 (33) | 49 (42) |
| Other | 0 (0) | 0 (0) | 5 (1) | 5 (1) | 0 (0) | 0 (0) | 0 (0) | 0 (0) |

^a^Includes five European countries only: UK, France, Germany, Spain, and Italy.

mCSPC, metastatic castration-sensitive prostate cancer; NHT, novel hormonal therapy; UK, United Kingdom; US, United States.

**Supplementary Table S3.** US insurance type by mCSPC treatment history.

| **US insurance type at time of data collection,**  ***n* (%)** | **Treated with taxane chemotherapy**  **(*n* = 12)** | **Treated with NHT**  **(*n* = 32)** | **No NHT and no taxane chemotherapy**  **(*n* = 72)** | **Total**  **(*n* = 116)** |
| --- | --- | --- | --- | --- |
| Medicaid^a^ | 1 (8) | 1 (3) | 8 (11) | 10 (9) |
| Medicare^b^ | 8 (67) | 20 (63) | 34 (47) | 62 (53) |
| Commercial insurance^c^ | 3 (25) | 7 (22) | 28 (39) | 38 (33) |
| Health insurance exchange plan | 0 (0) | 2 (6) | 1 (1) | 3 (3) |
| Tricare/Veterans healthcare | 0 (0) | 1 (3) | 0 (0) | 1 (1) |
| Other | 0 (0) | 0 (0) | 1 (1) | 1 (1) |
| No insurance coverage | 0 (0) | 1 (3) | 0 (0) | 1 (1) |

^a^Or state-specific equivalent.

^b^Including Medicare, Medicare Part D, Medicare medical savings account and Medicare Advantage.

^c^Including employer provided, partners’ employer or privately arranged.

mCSPC, metastatic castration-sensitive prostate cancer; NHT, novel hormonal therapy; UK, United Kingdom; US, United States.

**Supplementary Table S4.** mCSPC and mCRPC treatment trends by ethnicity across five European countries and the US.

|  | **Europe**^a^ | | | | **US** | | |
| --- | --- | --- | --- | --- | --- | --- | --- |
|  | **White/ Caucasian**  **(n = 564)** | **Afro-Caribbean**  **(n = 17)** | | **Other**^b^  **(n = 25)** | **White/ Caucasian**  **(n = 71)** | **African American**  **(n = 29)** | **Other**^b^  **(n = 16)** |
| **First-line mCRPC treatment, *n* (%)** | | |  | | | | |
| NHT + ADT^d^ (no taxane chemotherapy) | 372 (66) | 11 (65) | | 13 (52) | 55 (77) | 21 (72) | 11 (69) |
| Taxane chemotherapy + ADT^c^ (no NHT) | 147 (26) | 5 (29) | | 7 (28) | 6 (8) | 2 (7) | 2 (13) |
| Taxane chemotherapy + NHT + ADT^c^ | 8 (1) | 0 (0) | | 2 (8) | 0 (0) | 0 (0) | 0 (0) |
| ADT^c^ | 27 (5) | 6 (1) | | 2 (8) | 6 (8) | 4 (14) | 2 (13) |
| Other^d^ + ADT^c^ | 10 (2) | 0 (0) | | 1 (4) | 4 (6) | 2 (7) | 1 (6) |
| **mCSPC treatment, *n* (%)**^e^ | | | | | | | |
| Treated with NHT | 74 (13) | 0 (0) | | 2 (1) | 19 (27) | 11 (38) | 2 (13) |
| Treated with taxane chemotherapy | 91 (16) | 3 (18) | | 4 (16) | 8 (11) | 3 (10) | 1 (6) |
| No NHT and no taxane chemotherapy | 401 (71) | 14 (82) | | 19 (76) | 44 (62) | 15 (52) | 13 (81) |

^a^Includes five European countries only: UK, France, Germany, Spain, and Italy.

^b^Other ethnicities include: Asian (Indian subcontinent); Asian (other); Hispanic/Latino; Middle Eastern; and mixed race.

^c^ADT with or without a first-generation NSAA.

^d^Other treatment may include: abiraterone + enzalutamide, bisphosphonates, cabazitaxel + docetaxel, cisplatin, diethylstilbestrol, paclitaxel + carboplatin, radium-223-containing regimen (no sipuleucel-T), sipuleucel-T containing regimen (no radium-223).

^e^In Europe, 2 patients were treated with both taxane chemotherapy and NHT in mCSPC.

ADT, androgen deprivation therapy; mCRPC, metastatic castration-resistant prostate cancer; mCSPC, metastatic castration-sensitive prostate cancer; NHT, novel hormonal therapy; UK, United Kingdom; US, United States.

**Supplementary Table S5.** mCSPC and mCRPC treatment trends by physician specialty across five European countries and the US.

|  | **Europe**^a^ | | | **US** | |
| --- | --- | --- | --- | --- | --- |
|  | **Urologist**  **(n = 77)** | **Oncologist**  **(n = 529)** | | **Urologist**  **(n = 20)** | **Oncologist**  **(n = 96)** |
| **First-line mCRPC treatment, *n* (%)** | | |  | | |
| NHT + ADT^b^ (no taxane chemotherapy) | 49 (64) | 347 (66) | | 16 (80) | 71 (74) |
| Taxane chemotherapy + ADT^b^ (no NHT) | 11 (14) | 148 (28) | | 0 (0) | 10 (10) |
| Taxane chemotherapy + NHT + ADT^b^ | 4 (5) | 6 (1) | | 0 (0) | 0 (0) |
| ADT^b^ | 7 (9) | 23 (4) | | 1 (5) | 11 (11) |
| Other^c^ + ADT^b^ | 6 (8) | 5 (1) | | 3 (15) | 4 (4) |
| **mCSPC treatment, *n* (%)**^d^ | | | | | |
| Treated with NHT | 7 (9) | 69 (13) | | 2 (10) | 30 (31) |
| Treated with taxane chemotherapy | 14 (18) | 84 (16) | | 0 (0) | 12 (13) |
| No NHT and no taxane chemotherapy | 56 (73) | 378 (71) | | 18 (90) | 54 (56) |

^a^Includes five European countries only: UK, France, Germany, Spain, and Italy.

^b^Other treatment may include: abiraterone + enzalutamide, bisphosphonates, cabazitaxel + docetaxel, cisplatin, diethylstilbestrol, paclitaxel + carboplatin, radium-223-containing regimen (no sipuleucel-T), sipuleucel-T containing regimen (no radium-223).

^c^In Europe, 2 patients were treated with both taxane chemotherapy and NHT in mCSPC.

^d^ADT with or without a first-generation NSAA.

ADT, androgen deprivation therapy; mCRPC, metastatic castration-resistant prostate cancer; mCSPC, metastatic castration-sensitive prostate cancer; NHT, novel hormonal therapy; UK, United Kingdom; US, United States.

**Supplementary Table S6.** mCSPC and mCRPC treatment trends by US insurance status.

|  | **Medicare**  **(n = 62)** | **Commercial**  **(n = 41)** | | **Medicaid**  **(n = 10)** | **Other/no health insurance**  **(n = 3)** |  |
| --- | --- | --- | --- | --- | --- | --- |
| **First-line mCRPC treatment, *n* (%)** | | |  | | | |
| NHT + ADT^a^ (no taxane chemotherapy) | 44 (71) | 34 (83) | | 8 | 1 |  |
| Taxane chemotherapy + ADT^a^ (no NHT) | 4 (6) | 4 (10) | | 0 | 2 |  |
| ADT^a^ | 9 (15) | 1 (2) | | 2 | 0 |  |
| Other^b^ + ADT^a^ | 5 (8) | 2 (5) | | 0 | 0 |  |
| **mCSPC treatment, *n* (%)** | | | | | |  |
| Treated with NHT | 20 (32) | 9 (22) | | 1 | 2 |  |
| Treated with taxane chemotherapy | 8 (13) | 3 (7) | | 1 | 0 |  |
| No NHT and no taxane chemotherapy | 34 (55) | 29 (71) | | 8 | 1 |  |

^a^ADT with or without a first-generation NSAA.

^b^Other treatment may include: abiraterone + enzalutamide, bisphosphonates, cabazitaxel + docetaxel, cisplatin, diethylstilbestrol, paclitaxel + carboplatin, radium-223-containing regimen (no sipuleucel-T), sipuleucel-T containing regimen (no radium-223).

ADT, androgen deprivation therapy; mCRPC, metastatic castration-resistant prostate cancer; mCSPC, metastatic castration-sensitive prostate cancer; NHT, novel hormonal therapy; US, United States.
